# An Interesting Case of Dengue Encephalitis With Parkinsonism Sequela

**DOI:** 10.7759/cureus.44970

**Published:** 2023-09-10

**Authors:** Kiran Kuraning, Venkatesha Gupta KV, Pooja Murthy, Ajith Kumar AK, Nikhil N, Ganaraja V H

**Affiliations:** 1 Critical Care Medicine, Aster Hospital Whitefield, Bengaluru, IND; 2 Neurology, Vydehi Institute of Medical Sciences & Research Center, Bengaluru, IND

**Keywords:** arboviral illness, parkinsonism, double doughnut sign, encephalitis, dengue

## Abstract

Expanded dengue syndrome has been associated with various neurological manifestations. In this report, we present a rare and interesting case of parkinsonism secondary to dengue encephalitis in a young female. A 25-year-old female was admitted to the ICU with high-grade fever, generalized weakness, and altered sensorium for two days. Meningoencephalitis workup was negative but she tested positive for dengue non-structural 1 (NS1) antigen. MRI brain showed a “double doughnut sign” consistent with viral encephalitis. She was managed with neuroprotective measures, a short course of steroids, and invasive mechanical ventilation via tracheostomy. During the course of her treatment, she developed left upper limb rigidity, involuntary movements, and gait abnormalities with generalized bradykinesia suggestive of dengue-associated parkinsonism. She responded well to trihexyphenidyl and levodopa with significant neurological recovery and was discharged from the hospital in a condition where she could independently walk with significant improvement in dystonia. Central nervous system involvement has been well documented in arboviral illnesses caused by neurotropic viruses. Dengue encephalitis with sequelae like parkinsonism is potentially treatable when identified appropriately and in a timely manner.

## Introduction

Dengue infection is caused by the dengue virus (DENV), which belongs to the genus Flavivirus within the Flaviviridae family. DENV has four distinct serotypes: DENV-1, DENV-2, DENV-3, and DENV-4 [1]. The presentation of DENV infection ranges from asymptomatic cases to fatal illnesses like multi-organ dysfunction syndrome. Various neurological manifestations have been reported as part of expanded dengue syndrome, the most common being encephalitis and encephalopathy [2]; other less common manifestations include brachial neuritis, optic neuritis, Guillain-Barré syndrome, myositis, opsoclonus-myoclonus syndrome, and parkinsonism [3]. While several viruses cause parkinsonism, all have some common features including bradykinesia, tremors, hypokinesia, mask-like facies, and abnormal gait. In this report, we discuss an interesting case of dengue encephalitis with parkinsonism which was managed successfully.

## Case presentation

**Figure 1:**
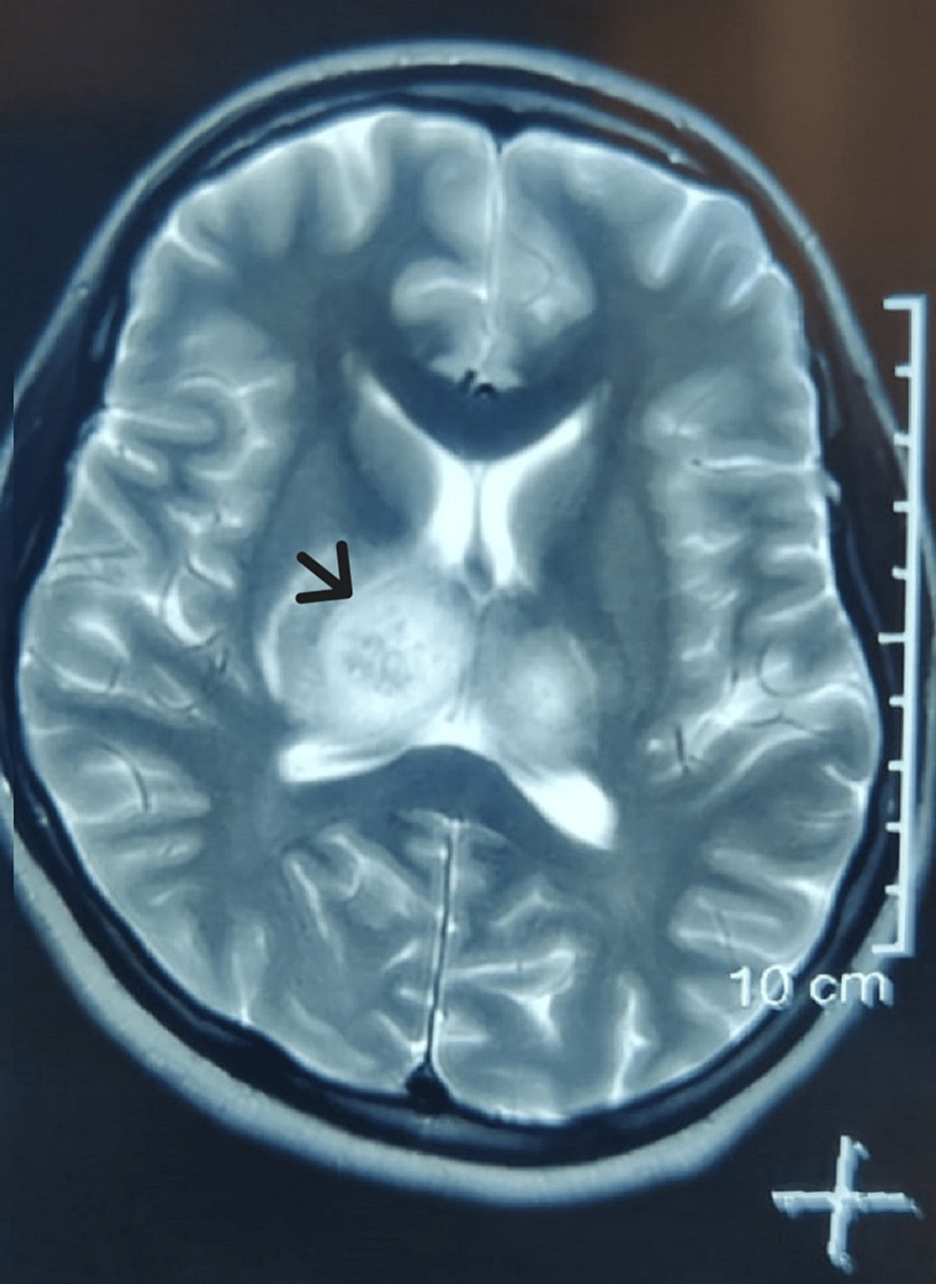
MRI brain showing double doughnut sign (arrow) MRI: magnetic resonance imaging

A 25-year-old female presented with complaints of high-grade fever, generalized weakness, and altered sensorium for two days. She was hemodynamically stable, and saturating 98% on room air. Central nervous system examination did not reveal any focal neurological deficits. She was admitted to the ICU in light of altered sensorium and excessive agitation. CT brain was normal and CSF analysis, including biochemistry, cell type, cell count, rapid molecular meningitis panel, CBNAAT (GeneXpert) for tuberculosis, as well as cultures were normal. Electroencephalogram (EEG) was suggestive of diffuse brain dysfunction and no seizure activity. Laboratory investigations revealed a decreasing trend of platelets, high hematocrit, transaminitis, and positive dengue NS1 antigen. She was managed with intravenous fluids, acetaminophen, N-acetyl cysteine infusion, and other supportive care along with close clinical and hematological monitoring. Prophylactic antiepileptic (levetiracetam) was administered due to a suspicious episode of seizure-like activity during the previous hospital visit. In light of her persistent altered sensorium, an MRI brain was done after 72 hours, which showed bilateral thalamic diffusion restriction (right > left), i.e., “double doughnut sign” with mild mass effect (Figure 1). These MRI findings were consistent with viral encephalitis. Based on the patient's history of fever, labs showing thrombocytopenia, and positive NS1 antigen in serum, a diagnosis of dengue encephalitis was established.

The patient received appropriate care including anti-edema measures. A repeat MRI performed on day eight in view of neurological worsening showed similar findings. She was intubated for airway protection and ventilated mechanically. Early percutaneous tracheostomy was performed in view of the anticipated prolonged time for neurological recovery. She received a course of intravenous dexamethasone (8mg Q8H) for three days. She also required multiple doses of quetiapine (starting with 12.5 mg 12th hourly, increased to 50 mg 12th hourly for a total of five days) and haloperidol (2.5 mg stat doses as required) in light of excessive agitation. On day 12 of illness, the patient developed rigidity of the left upper limb, involuntary movements of both upper limbs, and gait abnormalities. The possibility of drug-induced extrapyramidal symptoms was considered, and trihexyphenidyl and levodopa were added while the clinical response was monitored. Although differentiating disease-induced parkinsonism from the drug-induced variant is challenging, in light of MRI findings consistent with dengue encephalitis, disease-associated parkinsonism was considered the more appropriate diagnosis. However, antipsychotic medication could have aggravated the entire episode. She responded well to an escalated dose of trihexyphenidyl. She experienced gradual improvement In a week’s time, following which she was decannulated, and Ryle’s tube was discontinued. In the next two to three days, she was observed to have hoarseness of voice and features of microaspiration were noticed. Left vocal cord paralysis was noted with the aid of a flexible fiberscope, which required the re-insertion of Ryle’s tube. She underwent regular sessions of physiotherapy including speech and swallow therapy. After significant neurological recovery, she was discharged from the hospital on day 30 in a condition where she could independently walk with significant improvement in dystonia.

## Discussion

Dengue illness remains a major cause of morbidity and mortality in tropical and subtropical countries. The financial burden of the disease is significant, including medical costs and mosquito control costs [4]. Severe dengue involves multi-organ dysfunction and is also a major cause of death resulting from plasma leakage, fluid accumulation causing respiratory distress, severe hemorrhage, or organ failure [5]. Neurological complications of dengue can be categorized into dengue encephalopathy, encephalitis, neuromuscular complications, and neuro-ophthalmic involvement [6]. While neurological involvement in arboviral illnesses has been described extensively, movement disorders following these illnesses are rare. There are very few reports of various movement disorders associated with dengue encephalitis, the most common being parkinsonism, opsoclonus-myoclonus syndrome, and ataxia. A comprehensive search of existing literature (PubMed: dengue parkinsonism) revealed a total of seven case reports of dengue encephalitis-associated parkinsonism to date. Viral parkinsonism resembles idiopathic Parkinson’s disease with features like bradykinesia, tremor, hypokinesia, mask-like facies, tremor, postural instability, rigidity, and abnormal gait. Viral parkinsonism is induced by various viruses, including Japanese encephalitis virus, West Nile virus, HIV, Coxsackie virus, herpes simplex virus, varicella-zoster virus, cytomegalovirus, and DENV [7].

The criteria for recognizing DENV-associated parkinsonism involve a clinical diagnosis of a DENV infection with laboratory confirmation and a diagnosis of parkinsonism either during or immediately following dengue infection [8]. In our case, the patient had high-grade fever with thrombocytopenia, positive serum dengue NS1 antigen, and she developed parkinsonism following dengue infection, which fits the aforementioned criteria. Although brain imaging changes are not specific to dengue, they help to narrow down the differential diagnosis.

The actual mechanism of dengue infection on the central nervous system is still a matter of debate. Probable mechanisms of post-dengue parkinsonism are direct neurotrophic effect, vasculopathy, autoimmune phenomenon, and metabolic imbalance [8,9]. As per immunohistochemistry studies, various potent viruses are responsible for Lewy body formation and later cell death in the nigral region as in idiopathic parkinsonism. Hence, routine antiparkinsonian drugs may be effective in some such cases [4]. The absence of DENV in CSF in many case reports highlights that there can be other mechanisms than neurotropism. Such cases respond well to corticosteroids where the mechanism is likely to be autoimmune. Our patient responded well to trihexyphenidyl, an anticholinergic agent used in Parkinson’s disease. Attributing the cause here to anti-psychotic medication is difficult. Irrespective of the mechanism and modality of treatment for post-dengue parkinsonism, most cases witnessed improvement within two to seven weeks of onset [7]. A recent systemic review and meta-analysis did not prove the usefulness of steroids in viral encephalitis, but the study did not include patients with dengue. Further studies are needed to understand the pathophysiology of neurological involvement in dengue infection, which will help to devise definitive management strategies.

## Conclusions

Dengue-associated parkinsonism is rare but a known complication among various movement disorders reported as sequelae of dengue encephalitis. Differentiating disease-induced parkinsonism from the drug-induced variant is always challenging. The condition is completely curable If recognized and treated promptly. The treatment is mostly symptomatic.
